# Definition and Validation of an Exposure Measurement Method for a Typical Load of a Base Station

**DOI:** 10.1002/bem.70029

**Published:** 2025-11-01

**Authors:** Anna‐Malin Schiffarth, Tam Julian Ta, Christian Bornkessel, Lisa‐Marie Schilling, Matthias Hein, Dirk Heberling

**Affiliations:** ^1^ Institute of High Frequency Technology RWTH Aachen University Aachen Germany; ^2^ RF & Microwave Research Laboratory Thuringian Center of Innovation in Mobility, TU Ilmenau Ilmenau Germany; ^3^ Fraunhofer Institute for High Frequency Physics and Radar Techniques Wachtberg Germany

**Keywords:** 5G massive‐MIMO, exposure measurement, future exposure, typical utilization rates

## Abstract

In the context of risk communication in mobile radio, a discussion has emerged whether exposure at common load of a base station should be presented in addition to the theoretical maximum, especially with 5G massive‐MIMO. However, a reproducible measurement method for instantaneous exposure independent of the utilization of the cell has yet to be developed. To fill this gap, 10 mobile phone use cases were identified, the corresponding data rates were measured and categorized into low (20 Mbps), medium (200 Mbps), and high (600 Mbps) data rates to generate a typical base station load. A measurement method was developed, using iPerf on a user equipment to generate the data rates at a measurement point, while a channel power measurement is used to determine the exposure of all mobile radio services installed. The method was validated at four base stations, considering various factors such as reproducibility in relation to the number of users in the cell, averaging time, and application buffering. The results show the reliability of the method across different times of day and base station loads and that averaging over 30 sweeps provides reproducible exposure results. Consequently, this study presents a validated approach for measuring typical instantaneous exposure in real‐world mobile network conditions.

## Summary

- A novel method was developed to measure instantaneous exposure at typical base station loads using three representative data rates (low, medium, and high), providing a more realistic assessment of mobile phone exposure beyond maximum load conditions.
- The method was shown to be effective at different network load conditions and times of day, with consistent results over varying numbers of users.
- The study showed that maintaining a constant data rate through iPerf closely matched the exposure levels of actual applications, demonstrating the applicability of the method for risk communication in mobile communications.

## 1 Introduction

The continuous expansion of 5G networks, particularly in the sub‐6 GHz frequency range, and the increased demand for higher data rates from users has been a significant driver of the widespread deployment of massive‐MIMO technology. This technology offers advanced functionalities such as beamforming, which serves to enhance network efficiency by optimizing the capacity within the cells. As the deployment of 5G NR progresses, especially around the 3.6 GHz frequency, there is an increasing need to assess the exposure levels of electromagnetic fields in the vicinity of the base stations. Recent research has focused on the measurement of the maximum possible exposure, as the deployment of these base stations must comply with the human exposure limits set by the The International Commission on Non‐Ionizing Radiation Protection (ICNIRP) (1998) or its country‐specific implementations.

While much of the existing research focuses on the maximum possible exposure as summarized by Fellan and Schotten (2022), there is also a growing interest in measuring the actual exposure (Rybakowski et al. 2023) and an exposure during a typical load of a base station (Schiffarth and Heberling 2023). Numerous measurement campaigns have been performed in recent years, which have proven that maximum exposure in the vicinity of 5G massive‐MIMO base stations can be significantly higher than with previous technologies (e.g., GSM, UMTS, and LTE) due to the higher signal bandwidth and maximum gain (Kopacz et al. 2021; Deprez et al. 2025; Villaescusa‐Tebar et al. 2024). In contrast, exposure during common user activities, such as streaming a TV channel on a user equipment (UE) or in idle mode, is proportionally lower than the maximum possible exposure (Loh et al. 2021). Another aspect of 5G massive‐MIMO, in addition to the significant differences between the maximum exposure and the exposure during common user activities, is the possibility of beamforming. In this regard, research has shown that the exposure of a passive exposed person, that is, a person not using an own 5G UE, in a massive‐MIMO cell depends strongly on the location of active users due to beamforming capabilities (Kopacz et al. 2021; Heliot et al. 2022). Other studies have explored the stochastic nature of the exposure distribution through simulations and measurements, examining the probability of a full‐gain beam targeting at a single user in a cell (Jiang and Skrivervik 2022; Villaescusa‐Tebar et al. 2024; Galego 2024).

As part of efforts to enhance risk communication with the general public, it is of significant benefit to determine the actual maximum exposure of massive‐MIMO mobile radio stations and the exposure at a typical load of a base station. The former is discussed in greater detail by Xu et al. (2022) and Colombi et al. (2019), while the present research thoroughly investigates common usage scenarios, their associated data rates and exposure levels, and establishes a reproducible measurement procedure for these exposure levels. To establish a reliable methodology, first investigation were conducted at a base station in Aachen, Germany (Schiffarth et al. 2024). Here, 10 typical applications within a 5G cell were identified and their corresponding data rates determined. Based on these findings, three representative data rates were derived, which were then utilized to develop and evaluate a measurement methodology. The reproducibility of the exposure and the averaging time were assessed as key parameters in this process.

As the investigation by Schiffarth et al. (2024) only includes measurements in the vicinity of one base station, which was not heavily utilized, the investigations were extended here to three further base stations, which exhibit significant fluctuations in their load throughout the day. Furthermore, the impact of buffering on exposure is examined, comparing the instantaneous exposure with buffering and continuously downloading the corresponding data at a constant data rate. Additionally, tests to derive representative data rates for generating a common base station load were extended to more recent applications and retested with today's higher base station utilizations.

Section 2 outlines the methodology employed to determine typical use cases and corresponding data rates. It also presents the derivation of the three data rates utilized to generate a typical base station load. In Section 3, the measurement procedure for recording exposure at a typical base station load is presented. Moreover, the procedure and results of the measurement for verifying the reproducibility of the exposure and deriving an optimal averaging time are described. In Section 4, a conclusion is drawn.

## 2 Definition of Typical Usage Scenarios and Data Rates

The initial step was to derive data rates to generate a typical load of a base station for both current and future mobile radio applications. The basic methodology described by Schiffarth et al. (2024) was employed for this purpose and was extended by the inclusion of more contemporary applications. The method is outlined in the subsequent text. A total of nine usage scenarios for a typical mobile user were identified, including social media applications, download activities, streaming, and online gaming. Furthermore, a speed test serves as a reference for the maximum achievable data rate at the measurement point location. Table 1 lists the usage scenarios on the left. Other applications, such as telephoning, chatting, and video calling, were not included in the study as previous studies indicated that the data rates utilized for these activities were comparatively low or negligible and therefore still fall into the lowest group even when applying a worst‐case estimate.

**Table 1:** Examined usage scenarios and corresponding average, maximum, and minimum data rates across all measurement points.

Usage scenario	Average mean data rate (Mbps)	Maximum data rate (Mbps)	Minimum data rate (Mbps)
Social Media Browsing (TikTok)	0.4	0.8	0.1
TV‐Livestream (ARD)	4.0	5.3	1.3
Cloudgaming (PUBG Mobile)	4.9	20.0	0.1
Streaming (Amazon Prime)	7.2	8.0	5.6
Streaming (Disney+ IMAX format)	7.9	11.1	2.7
Live streaming video portal (Twitch)	7.9	12.0	5.3
Streaming (Netflix)	8.9	12.0	6.6
Streaming 8k Video (Youtube)	27.7	48.0	16.4
Download series (Netflix)	610.9	855.4	100.0
Speed test (FAST)	1090.6	1586.7	794.7

To ascertain the corresponding data rates, ten measurement points were selected in the vicinity of two massive‐MIMO base stations from the antenna manufacturer Huawei. The base stations are equipped with multiple LTE bands, including 900 MHz, 1.8 GHz, 2.1 GHz, and 2.6 GHz, as well as 5G in the 3.6 GHz and 2.1 GHz bands. In this context, 5G is operated in non‐standalone (NSA) mode, which implies that a 5G user depends on an LTE anchor band for core network operation. It is not feasible to mandate exclusive utilization of 5G without comprehensive intervention in the UE's programming. Consequently, a portion of the data rates was pulled via 5G and via LTE. The distribution of data rates between the two technologies depends on the measurement location relative to the base station, the manufacturer, and potentially also on the usage scenario or the load of the mobile cell.

To account for the variability of propagation conditions between the measurement point and the base station, the measurement points were systematically positioned across the cell, taking into account the azimuth angle relative to the boresight direction of the antenna, the distance from the base station, and the visual line of sight. The azimuth angles of the measurement points relative to the boresight direction of the antenna range from 0° to 57°, and the distance to the base station varies between 61 and 206 m. The diverse usage scenarios were run on a Samsung Galaxy S22 Ultra for a duration of 1 min each or for the duration of the download, with the UE positioned in a polystyrene holder on a tripod.

The data rate was calculated based on the amount of data utilized and the time required. The data rates thus determined for each usage scenario were subsequently averaged across all measurement points and grouped according to their respective data rates. The average mean, maximum, and minimum data rates for each usage scenario are presented in Table 1.

It can be observed that applications for social media, streaming and cloud gaming require low data rates, in the order of 10 Mbps and below. It should be noted that streaming 8k videos via YouTube is an exception to the above data rates and can therefore be placed in a second category. A third category comprises downloads with average data rates of 610 and 1091 Mbps.

Considering the aforementioned findings, three typical data rates (low, medium, and high) were selected. To accommodate higher video and gaming resolutions, the low data rates were rounded up based on the determined data rate per measurement point to 20 Mbps. In contrast, the data rates observed for the Netflix download and the speed test are highly variable, depending on the specific measurement point. These rates range from 100 Mbps to 1587 Mbps. To ensure that exposure can be measured at all measurement points regardless of the channel quality, the high data rate is rounded down to 600 Mbps. Furthermore, the downward estimation indicates that this measurement method can be employed even if the utilization of mobile communications systems increases in the future. Since the minimum recorded data rate for the Netflix download is 100 Mbps and the maximum recorded data rate for the 8k YouTube video is 48 Mbps, the average data rate is also rounded up to 200 Mbps in accordance with Schiffarth et al. (2024).

Consequently, data rates for a typical load of a base station of 20 Mbps (e.g., social media), 200 Mbps (e.g., streaming), and 600 Mbps (e.g., downloading series) were derived from these studies.

## 3 Development and Validation of a Method for Determining a Typical Exposure

### 3.1 Concept of a Reproducible Measurement Method

The objective is to develop a method for the reproducible measurement of the data rates derived in Section 2 and the instantaneous exposure resulting from these at a measurement point.

The first step was to develop a methodology for instigating the aforementioned data rates from the base station using a commercially available UE. The iPerf application 2024 was selected for this objective, as it enables the download of a specified data rate over a defined period via a server. A data repository search revealed a considerable number of publicly accessible iPerf3 servers 2024, where only one user is permitted at a time, thus allowing for the maximum data rate limited by the server.

The data rates (20, 200, and 600 Mbps, see Section 2) were then instigated in the vicinity of the measurement point via the iPerf application with a UE, while the instantaneous exposure was recorded using a channel power measurement. It is essential to record all 5G and LTE mobile radio bands available at the base station, due to the fact that a considerable number of networks are currently still operating in NSA. It is not possible to force the sole use of 5G in a UE with NSA networks.

Furthermore, 5G Eutra/NR Dual Connectivity (EN‐DC) is already utilized in numerous networks, where also further LTE carriers are employed for data acquisition, in addition to the LTE anchor band. In the long term, the concurrent utilization of 5G and 4G will become the standard scenario. Moving radio resources to other frequency bands for load balancing due to many users in the cell will also be common. Consequently, a joint assessment and output of the LTE and 5G instantaneous exposure is recommended.

Before performing the actual measurement, it is necessary to ascertain at each measurement point in the time domain that the exposure from the UE is less than that from the base station itself when a full buffer download is in use. This is mandatory for 5G massive‐MIMO base stations operating at 3.6 GHz, given that the downlink and uplink are transmitted on the same frequency in TDD format. It is therefore essential that the UE is positioned as close as possible to the measurement point so that the UE and the measuring device are reached by the same beam, while ensuring that it remains at a sufficient distance to guarantee that the uplink exposure is always lower than the downlink exposure.

In the following, this measurement method is evaluated with respect to the following points:

- Reproducibility of exposure with other users in the cell (Subsection 3.3).
- Influence of averaging time (Subsection 3.4).
- Effect of application buffering (Subsection 3.5).

### 3.2 Measurement Configuration

The Narda Selective Radiation Meter (SRM)‐3006 with the matching triaxial isotropic measurement probe was selected for use, with the “safety evaluation” mode enabled. The approach outlined by Bornkessel et al. (2021) was utilized to obtain the instantaneous exposure. The method is outlined in the subsequent text. In this mode, the instantaneous exposure of the various mobile radio standards, defined by the respective start and stop frequencies and the band‐specific resolution bandwidth (RBW), can be measured simultaneously. The safety evaluation mode is therefore comparable to the channel power measurements typically performed with a spectrum analyzer. The output includes both the individual exposures of the mobile radio bands and the total exposure. The sweep time, defined as the time required for the measuring device to deliver one exposure value per mobile radio band, depends on the total bandwidth to be measured and the RBW. The RBW is selected for each mobile radio band in such a way that Root Mean Square (RMS) smoothing of the fluctuating signal envelope due to digital modulation takes place. Therefore, to enable a valid comparison with the exposure limit values from ICNIRP (The International Commission on Non‐Ionizing Radiation Protection [ICNIRP] 1998) specified in RMS values, the average value was selected for the evaluation, resulting in a power‐related average value. The averaging interval was set to 6 min, but for measurement durations of less than 6 min, the average value is calculated across all exposures recorded so far. The measurement duration is discussed in more detail in Section 3.4. The spatial sweep method, where the probe is swept vertically through a test plane of 1 m^2^ at a height of 1.5 m while recording several sweeps, was used to account for locally varying fields.

During the validation of the measurement method described below, the data rates (Section 2) were configured using iPerf on a Samsung Galaxy S22 Ultra, while the UE was set to prioritize 5G usage. Preliminary investigations have shown that other smartphones generate comparable exposure within the extended measurement uncertainty of ±3 dB at the same provoked data rate.

Samsung smartphones usually allow access to the connection parameters via a so‐called service mode. This mode displays various connectivity information, including:

- The DL (downlink) and UL (uplink) frequencies of the connected anchor band;
- The connected NR band, the PCI of the connected cell, the data throughput via 5G only and via 4G only;
- The number of connected cells via Carrier Aggregation (CA) and Evolved Non‐Standalone Dual Connectivity (EN‐DC), as well as measurement data of neighboring cells (band, Physical Cell Identity [PCI], Received Signal Strength Indicator [RSRP], and Signal‐to‐Noise Ratio [SINR]).

As the service mode does not permit app access or storage of the data, an application has been developed to reliably read and store the service mode data. To achieve this, the smartphone is connected to a PC via USB and the application is started. Once the service mode has been recorded, all values are saved in a raw text file and sorted in an Excel spreadsheet.

In this way, the effects of different data rates or the use of only 4G without 5G on the instantaneous exposure could be identified retrospectively.

The first base station was chosen in the greater Aachen area at a base station equipped with Huawei technology so that initial findings and experience could be gathered and analyzed quickly, and measurements could be repeated if necessary.

In this study, three further base stations were selected in collaboration with the network operator, with the objective of providing a high degree of variability in the load of the 5G massive‐MIMO base station throughout the day. These included one massive‐MIMO base stations from Huawei (Cologne, Germany) and two from Ericsson (Darmstadt and Bruchköbel, Germany). The mobile phone bands available at each base station are listed in Table 2.

**Table 2:** Existing mobile phone bands (MB) at the tested base stations.

Base station	Center frequencies of mobile radio band (MHz)	Manufacturer massive‐MIMO‐antenna
Aachen	955, 1820, 2160, 2650, 3655	Huawei
Köln	955, 1820, 2160, 2650, 3655	Huawei
Darmstadt	816, 955, 1820, 2160, 2650, 3655	Ericsson
Bruchköbel	816, 955, 1820, 2160, 3655	Ericsson

None of the base stations had a power lock feature activated, which is a power adjustment system that regulates the transmission power on a 6‐min average to meet the exposure limit values. The results presented by Schiffarth et al. (2024) are also presented in the following sections for comparison with the new selected base stations.

**Table 3:** Listing of the recorded measurement points (MP) in Aachen (A1–A4), Cologne (C1–C4), Darmstadt (D1–D4), and Bruchköbel (B1–B4) including the line‐of‐sight condition, distance to the 5G base station (BS), azimuth angle to the boresight direction, and their position in relation to the 3 dB coverage area of the main lobe of the traffic antenna pattern.

MP	Distance MP‐BS (m)	Visual line of sight (LOS)	Azimuth angle to boresight direction (°)	In 3 dB coverage area of main lobe of traffic antenna pattern
A1	230	Yes	3	Yes
A2	157	No	28	No
A3	88	Yes	16	No
A4	128	No	42	No
C1	248	Yes	1	No
C2	152	No	71	No
C3	145	Yes	4	No
C4	327	No	19	Yes
D1	48	Yes	2	No
D2	58	Yes	32	No
D3	160	Yes	12	Yes
D4	232	No	18	Yes
B1	83	No	26	No
B2	183	Yes	9	Yes
B3	239	Yes	45	Yes
B4	266	No	0	Yes

**Figure 1:**
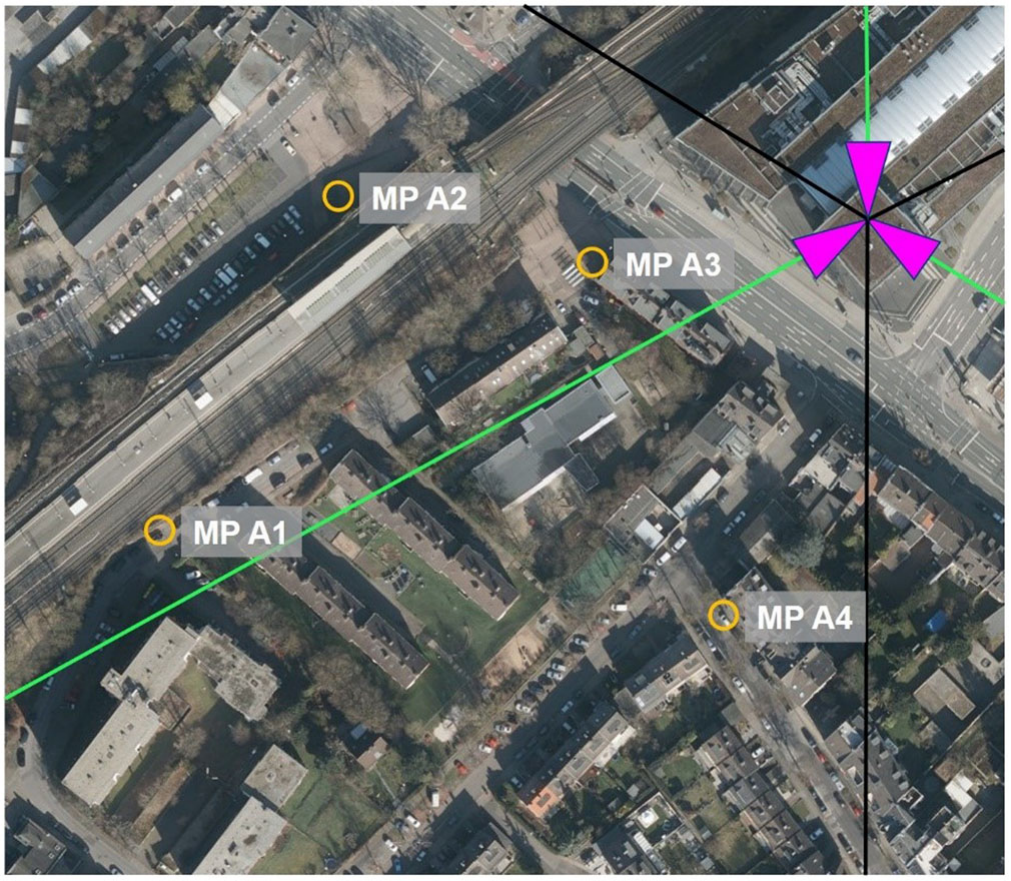
Distribution of measurement points (MP) A1–A4 for the validation measurements (yellow circles) in the vicinity of the 5G massive‐MIMO base station in Aachen (Geschäftsstelle des IMA GDI Nordrhein‐Westfalen 2025). The sector orientations are marked as magenta triangles, the main beam direction as green lines, and the cell boundaries as black lines.

**Figure 2:**
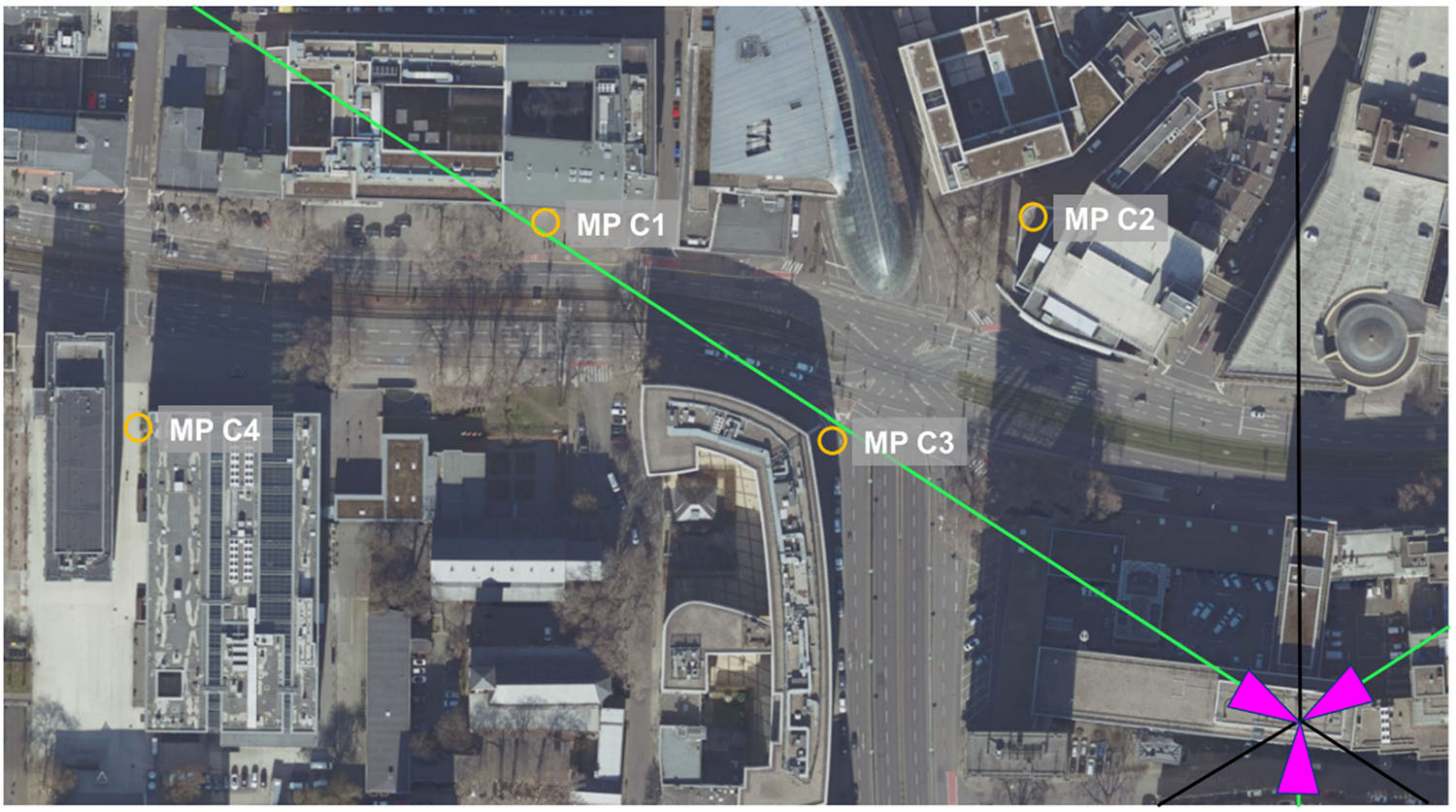
Distribution of measurement points (MP) C1–C4 for the validation measurements (yellow circles) in the vicinity of the 5G massive‐MIMO base station in Köln (Geschäftsstelle des IMA GDI Nordrhein‐Westfalen 2025). The sector orientations are marked as magenta triangles, the main beam direction as green lines, and the cell boundaries as black lines.

**Figure 3:**
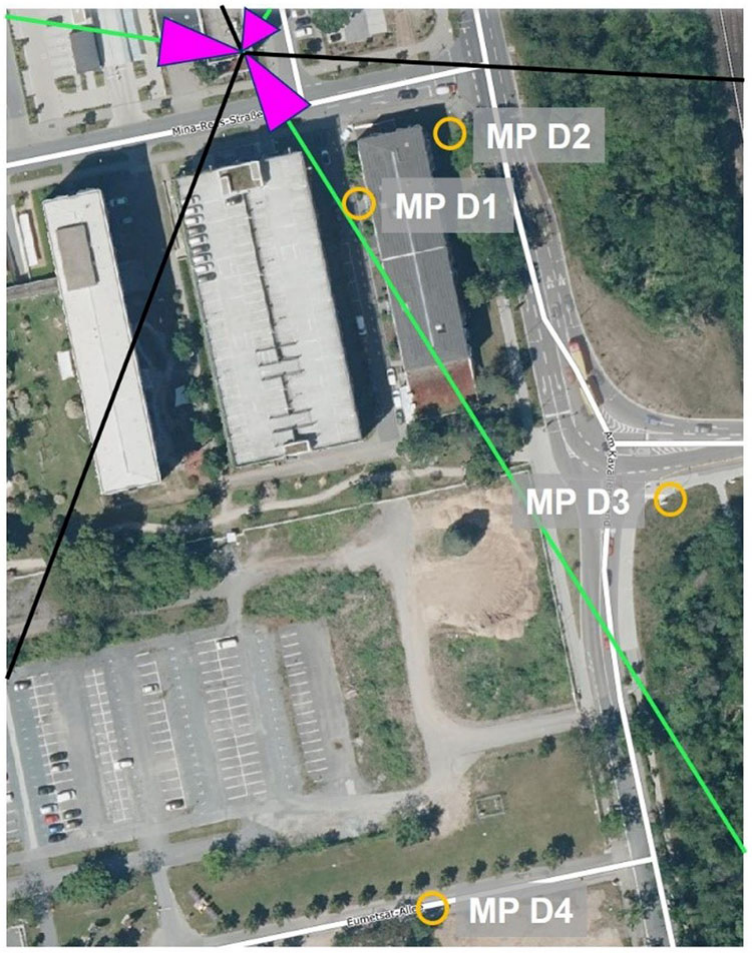
Distribution of measurement points (MP) D1–D4 for the validation measurements (yellow circles) in the vicinity of the 5G massive‐MIMO base station in Darmstadt (Geschäftsstelle des IMA GDI Hessen 2025). The sector orientations are marked as magenta triangles, the main beam direction as green lines, and the cell boundaries as black lines.

**Figure 4:**
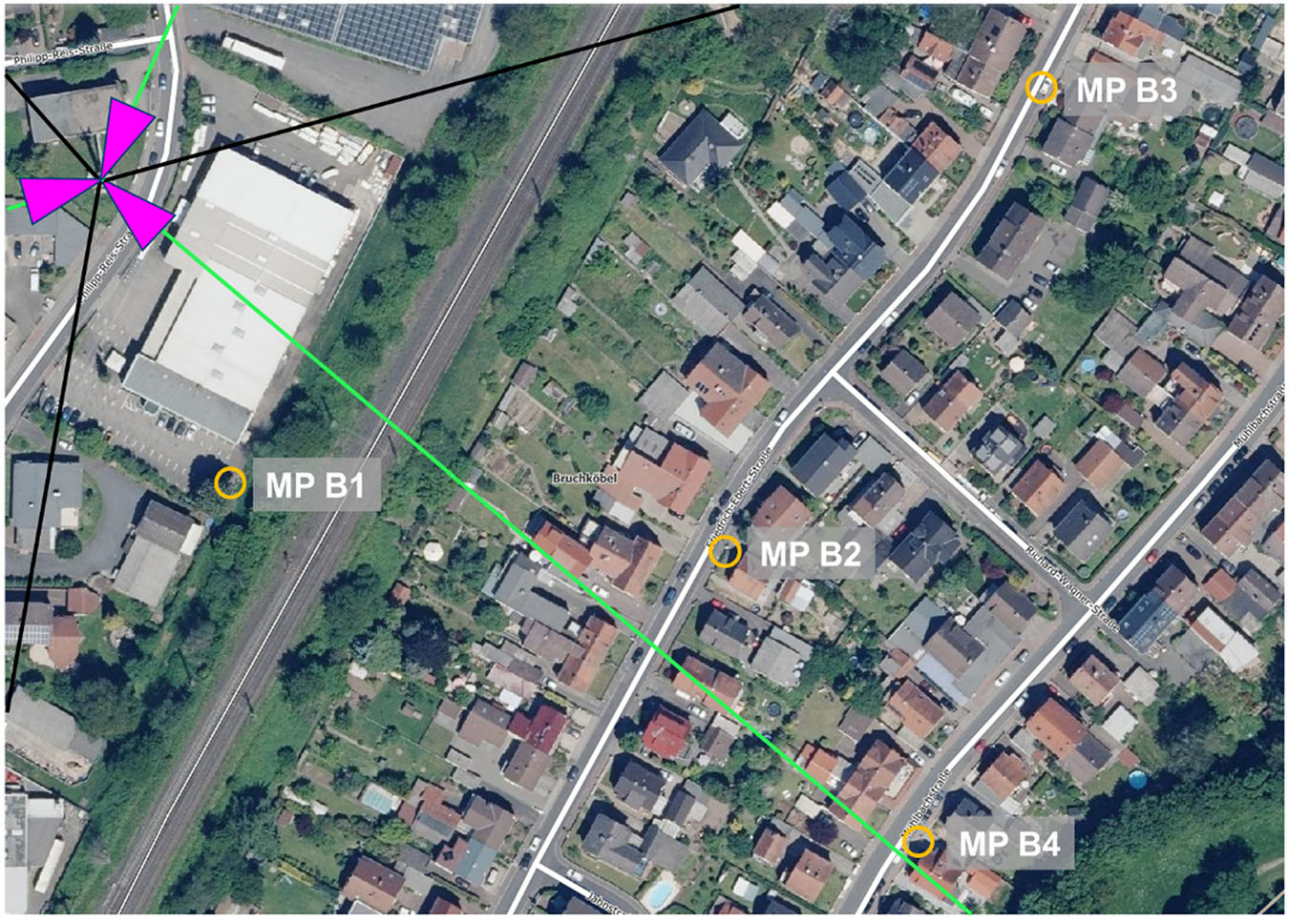
Distribution of measurement points (MP) B1–B4 for validation measurements (yellow circles) in the vicinity of the 5G massive‐MIMO base station in Bruchköbel (Geschäftsstelle des IMA GDI Hessen 2025). The sector orientations are marked as magenta triangles, the main beam direction as green lines, and the cell boundaries as black lines.

At each base station, four measurement points were chosen, varying in distance and the visual line of sight to the base station. Table 3 displays the distance to the base station, the visual line of sight condition, azimuth angle to the boresight direction, and position relative to the 3 dB coverage area of the main lobe of the traffic antenna pattern for each measurement point. The base stations and measurement points are shown in Figures 1, 2, 3, 4.

### 3.3 Reproducibility of the Exposure at a Typical Base Station Load

Since this measurement method should be used at all times and under usual operating conditions, it is important to repeat the measurement procedure under different base station operational conditions and compare them for reproducibility. The network operator determined the typical peak traffic hours for each base station in advance.

For this purpose, three measurement rounds M_1_–M_3_ were performed, which are supposed to reflect different load states of the mobile radio system:

- M_1_: Low utilization (after morning rush hour).
- M_2_: Medium utilization (during peak traffic hours).
- M_3_: High utilization (two other UEs were running a speed test at two of the other measurement points [horizontally or vertically at least one traffic beam width away]).

Throughout the measurements, it was ensured that all UEs used were connected constantly to the base station under investigation.

Following the measurements, monitoring data averaged over a period of 30 min showed that utilization of M_2_ was higher than utilization of M_1_ at all base stations. However, an exact correlation with individual measurement results could not be derived, as the monitoring data could only be read out at a time interval that was too coarse (every 30 min) for this purpose.

**Figure 5:**
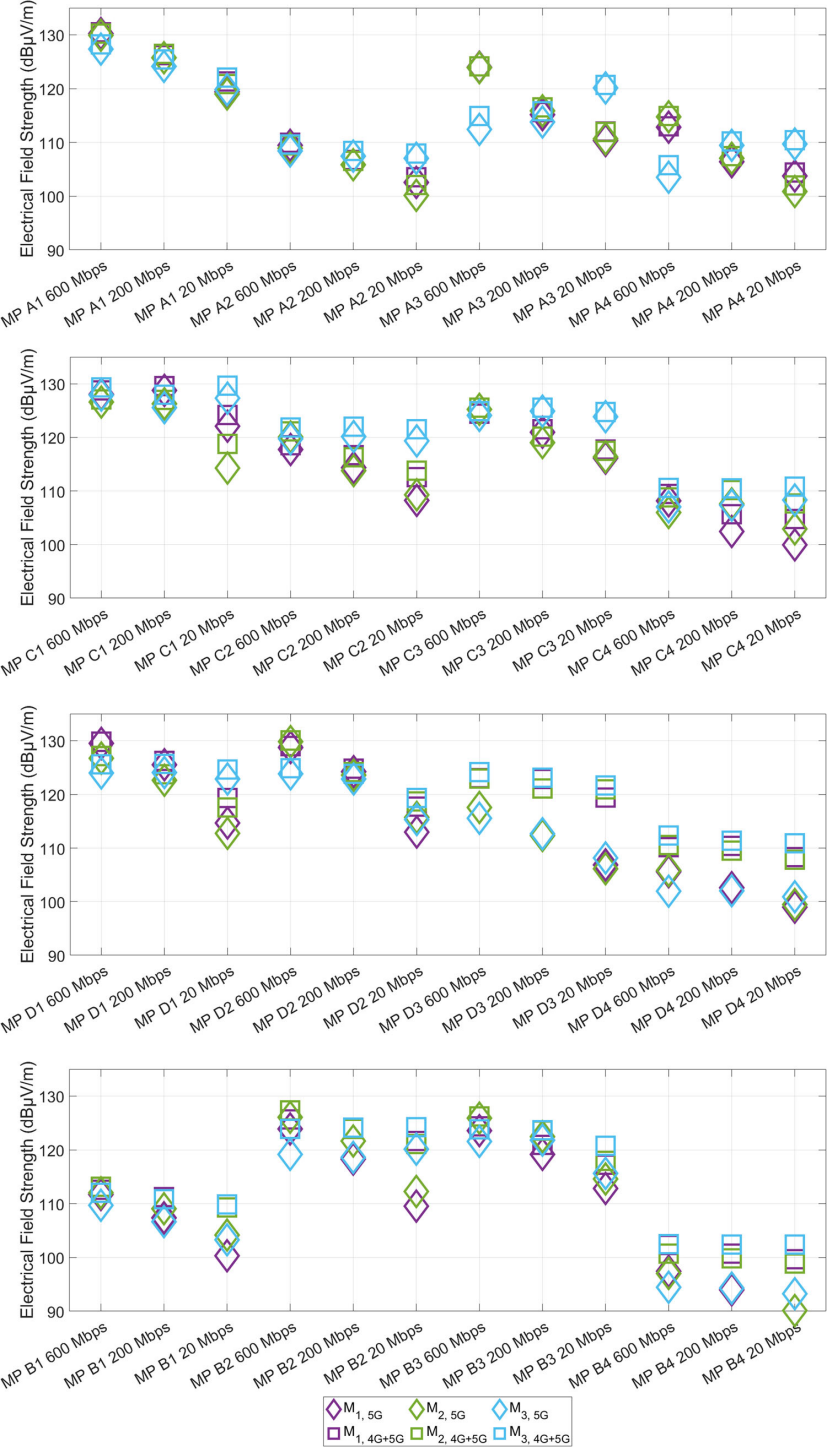
Comparison of 5G and total instantaneous exposure at the different measurement runs M_1_–M_3_ at the four measurement points at (from top to bottom) Aachen (A1–A4), Cologne (C1–C4), Darmstadt (D1–D4), and Bruchköbel (B1–B4).

Figure 5 illustrates the averaged instantaneous exposure at all measurement points per location at 20 Mbps, 200 Mbps, and 600 Mbps for M_1_ to M_3_. The instantaneous exposure was recorded over 30 sweeps, with measurement times ranging from 60 to 73 s, depending on the number of mobile radio bands present. The influence of the averaging time is further discussed in Subsection 3.4. For each measurement point and data rate, the results for the three load situations are depicted individually for 5G massive‐MIMO at 3.6 GHz only and the total combined exposure (5G + 4G).

As can be seen from the 5G and 4G + 5G markers, exposure to 5G significantly exceeds exposure to 4G in most cases. The 5G and total exposure obtained in measurement rounds M_1_ and M_2_ are reproducible within the extended measurement uncertainty of ±3 dB. As discussed in Subsection 3.1, it is currently not possible to enforce a UE to exclusively operate 5G. For this reason, the deviations in the total exposure are specifically addressed here. When the overall exposure is considered, it was observed that deviations outside the measurement uncertainty between M_1_ and M_2_ occurred only at two measurement points at one data rate each. At C1 at 20 Mbps, a deviation of 7.8 dB was noted due to a change in the measurement environment, with a truck being in the visual line of sight to the base station at M_2_. These findings indicate that when measuring a typical exposure, it is essential to ensure that the environment at the time of measurement accurately reflects typical conditions. A deviation of 4.9 dB was observed at C4 at 200 Mbps, where the self‐generated exposure was likely masked by another high‐data rate user. However, this could not be verified afterward. Between LOS and NLOS measurement points, no discernible difference could be identified.

Furthermore, it can be observed at several measurement points (A3, A4, D1, D2, B2, and B3) that in M_3_ (two other high‐throughput users in the cell) at 600 Mbps, the 5G exposure and the total exposure decrease significantly in comparison to M_1_ and M_2_. Due to the other high‐throughput users, sufficient resources can no longer be allocated to the measuring UE at the measurement point, which consequently results in a reduction of data rate and therefore also to a reduction in exposure. In contrast, at numerous measurement points at a data rate of 20 Mbps, it is evident that the self‐generated exposure at the measurement point was masked by the exposure of the other high‐data rate users, which were located at the neighboring measuring points. This is because when serving multiple users, the antenna pattern widens to supply multiple users via one beam or several individual beams, whereby these individual beams are also wider than a single aligned beam. Overall, this effect was observed in 18 of the 48 cases (four locations, four measurement points each, and three data rates each).

It can thus be concluded that the measurement method is effective across different times of day, under varying load conditions, and with a high number of other users. This is evidenced by the fact that only in two cases was a deviation of more than 3 dB between the total exposure levels found. In the case of two high‐throughput users in the cell, however, the measurement method was found to be of limited use, as it resulted in deviations of the total exposure exceeding 3 dB in a total of 18 out of 48 cases. It should be noted that it is highly unlikely that at least two other high data rate users will be continuously active over the entire measurement period (more than 1 min) in practice.

**Figure 6:**
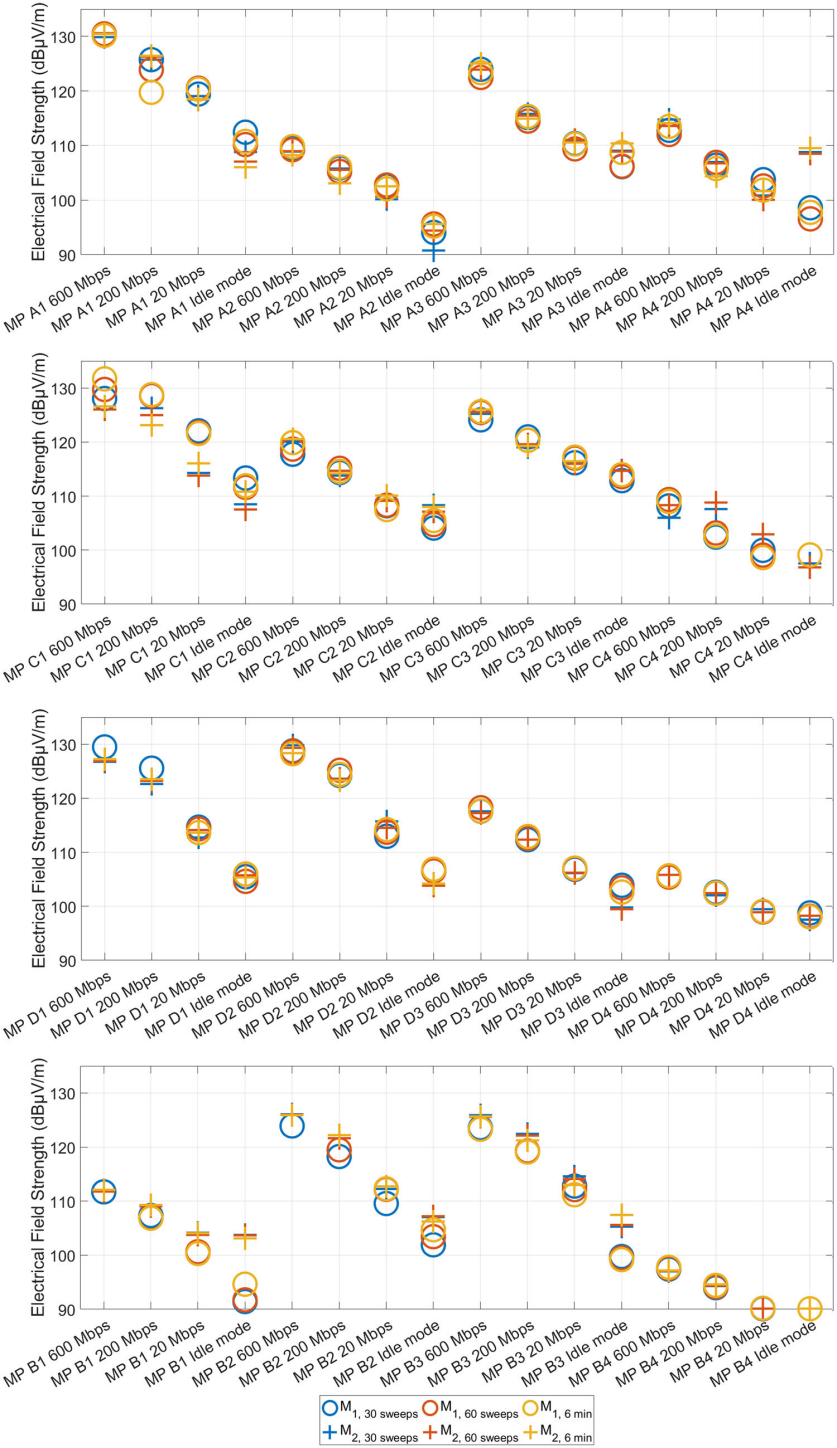
Comparison of the 5G exposures at the different averaging times per applied data rate at the four measurement points at (from top to bottom) Aachen (A1–A4), Cologne (C1–C4), Darmstadt (D1–D4), and Bruchköbel (B1–B4).

**Figure 7:**
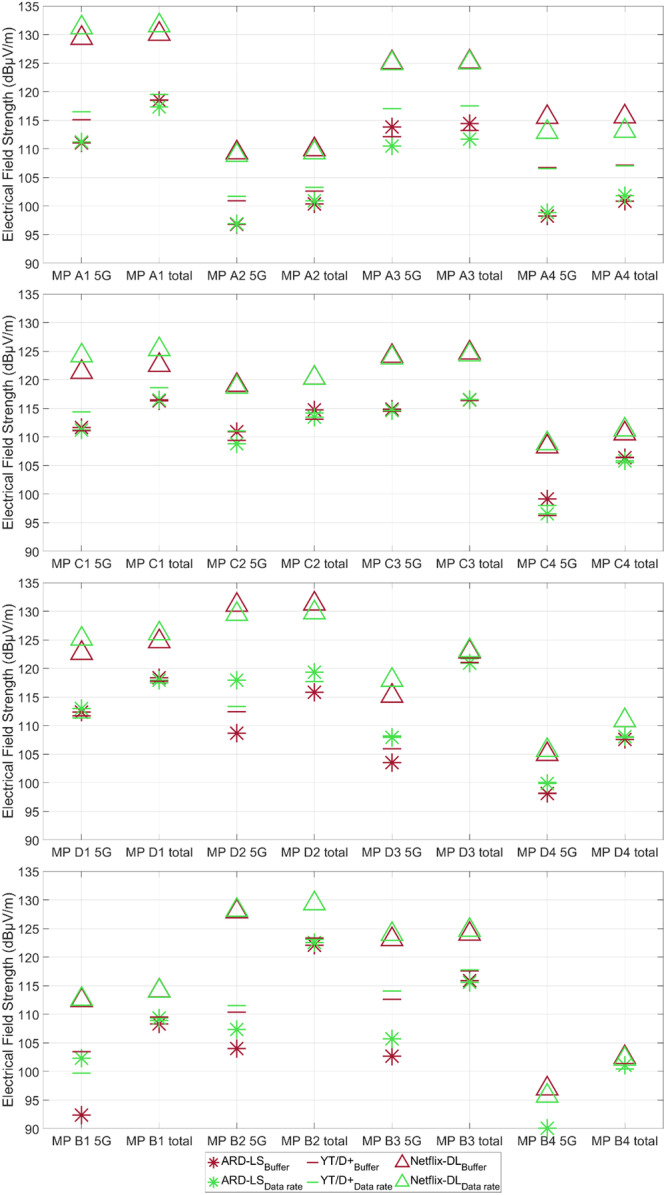
Comparison of 5G and total instantaneous exposure in the usage scenario with buffering and constant data rate with iPerf at the four measurement points at (from top to bottom) Aachen (A1–A4), Cologne (C1–C4), Darmstadt (D1–D4), and Bruchköbel (B1–B4).

### 3.4 Influence of Averaging Time

Time averaging is a critical factor in the assessment of exposure. The averaging time of 6 min, as used by Schilling et al. (2022), was the standard for obtaining the average value in accordance with the ICNIRP standard (The International Commission on Non‐Ionizing Radiation Protection [ICNIRP] 1998) until the publication of new guidelines by the ICNIRP in 2020, at which point an additional averaging time of 30 min was introduced. Nevertheless, the 1998 version continues to serve as the basis of many of the country‐specific regulations on electromagnetic exposure, for example, in Germany (ImSchV 2020). Conversely, Aerts et al. (2019) and Bornkessel et al. (2021) posit that a measurement interval of 1 min is adequate to account for local field strength fluctuations when using the sweeping method. To identify a short but still feasible averaging time that is nevertheless sufficient to account for the high variability of exposure resulting from beamforming at 5G, an investigation was conducted to determine the necessary measurement duration to obtain a reproducibly measurable exposure (deviation between exposure values of no more than ±3 dB).

Based on the findings of the investigations presented by Bornkessel et al. (2021), Aerts et al. (2019), and Schilling et al. (2022), a potential measurement time of 1 min was identified. Additionally, a measurement time of 2 min was selected. Due to the German implementation of the 1998 ICNIRP standard, a measurement time of 6 min was also set.

As the sweep time depends upon both the total channel bandwidth and the RBW, the initial measurements conducted at the base station in Aachen were employed to determine the number of sweeps that could be performed during the respective averaging time with the selected bands and settings. For the purposes of this study, the sweep counter of the SRM‐3006 was utilized to outline a measurement procedure that would allow the use of different sweep times at different base stations. The comparability of exposures is ensured through consistent measurement times for each mobile communications band and a constant data rate throughout the entire measurement process.

At the base station analyzed in Aachen, around 30 sweeps could be recorded in 1 min and 60 sweeps in 2 min. For better comparability of the measurements (same number of sweeps), it was decided to carry out the measurements with 30 and 60 sweeps also at the other base stations in Cologne, Darmstadt, and Bruchköbel. Due to the German legal situation regarding electromagnetic exposure, the value after 6 min was also recorded in addition to 30 and 60 sweeps.

Accordingly, to evaluate an appropriate averaging time, the probe of the SRM‐3006 was swept vertically through the test plane of 1 m^2^ at a height of 1.5 m for a period of 6 min, and the averaging time in the SRM‐3006 was set to 6 min. This measurement procedure was carried out twice for each measurement point when recording measurement rounds M_1_ and M_2_ (see Subsection 4.3). The exposure value was saved during the measurements after 30 sweeps and 60 sweeps and after 6 min.

In addition to the measurements at 20 Mbps, 200 Mbps, and 600 Mbps, the idle exposure was also recorded when the cell was utilized by other users only, that is, the own UE was in flight mode. This was carried out to evaluate whether background measurements of exposures caused by other users were reproducible as well.

Figure 6 shows the 5G exposure at different averaging times for each measurement point per base station for the measurement runs M_1_ and M_2_. M_3_ is not shown here, as only the value at 30 sweeps was recorded in the third measurement round. The remaining averaging times were not recorded for M_3_ due to the inability to run the two other UEs with Speedtest at the same data rate for the entire duration of the measurement. This was due to various issues, including heat problems of the UEs and dropouts from 5G to 4G. Nevertheless, other results would not be expected for this study, as in the case of two other UEs running a Speedtest, a constant high and consistent utilization of the cell is given. Since the results of the total exposure and the 5G exposure lead to the same findings, no visualization is provided here for better clarity.

Overall, the reproducibility of the measured values at different averaging times and even across multiple measurement runs (with two exceptions) is within the measurement uncertainty (±3 dB) at all base stations. Only MP A1 and MP C1 show a larger deviation (6.7 and 5.7 dB, respectively) due to a dropout from 5G to 4G of the mobile connection from the UE.

Furthermore, it is evident that at some measurement points (A4, B1, and B3) the instantaneous exposure in idle mode exhibits considerable fluctuations between measurement rounds and averaging times. With even higher utilization of the base station in the future, it can be assumed that this would be the case at all measurement points. Therefore, a direct measurement of the instantaneous exposure caused only by other users in the cell lacks reproducibility and is therefore unsuitable for inclusion in a reproducible measurement method of a typical exposure.

Also, a notable decrease of the instantaneous exposure was observed at all measurement points toward lower data rates. The lowest exposure was always observed in idle mode.

In conclusion, the findings of this investigation indicate that averaging over 30 sweeps is sufficient for obtaining a reproducible result of an instantaneous exposure while provoking a data rate close to the measurement point. Based on the data collected, averaging over 6 min appears to be disadvantageous, as there is a possibility that the UE may drop out of 5G during this time, or perform differently due to a highly variable environment. Furthermore, there is no evident advantage in averaging over 60 sweeps instead of 30 sweeps.

### 3.5 Influence of Buffering on the Exposure

Although typical data rates were derived in Section 2 using the averaged data rate of common use cases, this does not guarantee that such use cases and an equivalent constantly provoked data rate result in the same exposure. Possible deviations between those exposures may occur, particularly in applications involving buffering. Buffering is defined as the process of preloading data into a temporary storage area (buffer), with the objective of ensuring smooth playback or processing, particularly in the context of streaming media, such as videos or audio. Therefore, a comparison was conducted using three typical usage scenarios and their corresponding data rates.

The following procedure was applied: First, three usage scenarios were selected (one of each category, see Section 2), including TV live streaming of the German television channel ARD (ARD LS), Disney+ IMAX format/YouTube 4k video streaming (YT/D+), and downloading a series from Netflix (Netflix DL). Due to technical difficulties with the application, it was not possible to smoothly play a movie on Disney+ at some of the measurement points. Therefore, due to the comparable data rates (see Section 2), 4k video on YouTube was chosen as fall‐back solution.

In the case of ARD LS and YT/D+, the respective usage scenario was played for 30 sweeps (approximately 1 min; see Section 3.4 for the duration of the complete download in the case of Netflix DL (16–56 s), at each measurement point. During this time, the instantaneous exposure of all available 4G and 5G bands at the base station was measured. The required amount of data was read from the UE settings and the duration was recorded using a stopwatch. Consequently, the data rate was calculated based on the duration and the amount of data consumed at each measurement point individually. Next, this data rate was requested with iPerf while the corresponding exposure was recorded over 30 sweeps.

Figure 7 display the averaged exposures for the three applications and the provocation of the equivalent data rate with iPerf per location and per measurement point, divided into 5G exposure and total exposure (4G + 5G). When comparing exposures during buffering and constant data rate, it becomes evident that the 5G exposure and total exposure (4G + 5G) per usage scenario are in accordance with the measurement uncertainty of ±3 dB, with a few exceptions.

In total, there are only five instances of deviations of the 5G exposure greater than the expanded measurement uncertainty of ±3 dB out of a total of 48 cases (four base stations, four measurement points each, and three applications each). When examining the total exposure, there were only two deviations greater than 3 dB: at A3 using Disney+ IMAX format/YouTube 4k video streaming resulted in a deviation of 4.3 dB and at D2 using TV live stream in a deviation of 3.5 dB. Individual deviations cannot be traced in detail. The deviations may be due to changes in the distribution of data rates between 4G and 5G, measurement inaccuracies in the data rate, or fluctuations of the data rate obtained by iPerf. Therefore, a continuous monitoring of the UEs is recommended for future investigations so that further information is available, for example, on fluctuations of the provoked data rates, even if the deviations of the total exposure occurred only in 2 of 48 cases.

At most measurement points, the exposure to 5G for downloading a Netflix series was not significantly different from the total exposure, that is, most of the exposure is caused by the 5G massive‐MIMO base station. However, for the other two applications, there are partially large differences between the 5G exposure and the total exposure, suggesting that a significantly smaller part is due to 5G while the 4G exposure dominates. It is not possible to determine whether these higher 4G exposures are caused by other users in the cell or by the user's own data traffic.

The highest instantaneous exposure at all measurement points was observed for downloading the Netflix series. Depending on the measurement point, 1.38 or 1.89 GB were downloaded in 16–55 s, which corresponds to data rates of approximately 300–700 Mbps. The different file sizes are due to an app update, which allowed the series to be downloaded with a higher resolution. The TV livestream resulted in a data rate of 4 Mbps at each measurement point, while Disney+ IMAX format/YouTube 4k video streaming used data rates between 10 and 36 Mbps. Only Cologne Disney+ IMAX format/YouTube 4k video streaming experienced lower data rates of 1.5–10 Mbps, as both applications had problems playing the videos at every measurement point. Accordingly, Figure 7 clearly show that the 5G exposures for Disney+ IMAX format/YouTube 4k video streaming are higher than for live TV streaming, with the exception of the aforementioned measurement points C1–C4 in Cologne.

## 4 Summary and Conclusion

In the context of risk communication in mobile communications, a discussion has emerged regarding the potential merits of presenting an exposure at typical base station load, in addition to the maximum exposure. A measurement method that allows for the reproducible recording of this instantaneous exposure, irrespective of the number of users in the cell, has yet to be developed. For this reason, 10 usage scenarios were identified for a mobile phone user, and the associated data rate was determined at 10 measurement points. These results were averaged, grouped by data rate and three representative data rates to generate a typical load of a base station were derived: 20 Mbps (e.g., social media), 200 Mbps (e.g., streaming), and 600 Mbps (e.g., downloading series).

Subsequently, the measurement method was presented, whereby the three data rates are generated on a UE using iPerf in the vicinity of the measurement point, while a channel power measurement is used to determine the exposure of all mobile radio devices installed at the base station. The validation of the measurement method involved an assessment of its reproducibility in relation to the number of other users in the cell, the impact of averaging time, and the influence of an application's buffering.

Measurement rounds were conducted with low and high utilization by other users in the cell, as well as with the addition of two further high data rate users. From the results, it can be concluded that the measurement method is effective across different times of day, under varying load conditions, and with a high number of other typical users compared to the usual network load. In the case of two high‐throughput users in the cell, however, the measurement method was found to be of limited use, as it resulted in deviations in the total exposure exceeding 3 dB in a total of 18 out of 48 cases. It should be noted that it is highly unlikely that at least two other high‐throughput users will be continuously active over the entire measurement period (more than 1 min) in practice.

From the investigations regarding the optimal measurement or averaging time, it can be found that averaging over 30 sweeps is sufficient for a reproducible result of a typical instantaneous exposure while provoking a data rate close to the measurement point. Based on the data collected, averaging over 6 min appears to be disadvantageous, as there is a possibility that the UE may drop out of 5G during this time, or perform differently due to a highly variable environment.

A comparison between the instantaneous exposure of an actual application and the instantaneous exposure, when the corresponding constant data rate is provoked using iPerf, revealed that, overall, in only 5 out of the 48 cases examined, a higher deviation than the expanded uncertainty was observed in the comparison of the 5G exposures. Regarding the total exposure, a deviation of more than 3 dB was determined in only two of these cases. It thus follows that maintaining a constant data rate in iPerf is equivalent in terms of instantaneous exposure compared to typical usage scenarios, regardless of whether user data is buffered or not.

In summary, the studies presented here demonstrate the feasibility of measuring an instantaneous exposure at a typical base station load using the measurement method outlined, and subsequently outputting it for risk communication, in addition to the maximum possible exposure at each measurement point.

In the future, the integration of this measurement method for typical load conditions into the IEC 62232:2022 standard (International Electrotechnical Commission [IEC] 2022) could be discussed, as a method for deriving typical loads is already proposed there. In its present version, it mentions in‐situ measurements taken during regular network operation over a longer period of time without provoking a load condition. Additionally, exposure under a typical load condition is to be determined while streaming a video, livestreaming, or downloading a 1 GB data file. A consistent, reproducible measurement method by provoking a data rate over a relatively short period would be a useful addition.

## Ethics Statement

The authors have nothing to report.

## Conflicts of Interest

The authors declare no conflicts of interest.

## Data Availability

Part of the data that support the findings of this study are available from the corresponding author upon reasonable request.
